# Redefining water scarcity through the integrated water strategic resilience index amid climate and conflict pressures

**DOI:** 10.1038/s41598-026-42170-2

**Published:** 2026-03-14

**Authors:** Filippo Verre, Krishna Kumar, Ronny Berndtsson, Hossein Hashemi

**Affiliations:** 1 Centre for Advanced Middle Eastern Studies, Lund University, Lund, Sweden; 2 Division of Water Resources Engineering, Lund University, Lund, Sweden; 3 United Nations University Hub (UNU Hub) for Water in a Changing Environment, Lund University, Lund, Sweden

**Keywords:** Water resilience, Water scarcity, Climate change, Water security, Governance, Conflict impact, Environmental sciences, Environmental social sciences, Hydrology

## Abstract

Water scarcity is a dynamic condition influenced by a variety of factors, including environmental variables but also political, economic, technological, and social variables. This research reflects the intersection of natural resources, governance, and human systems. Redefining water scarcity is a crucial factor for greater sustainable management in the face of increasing climate variability and geopolitical stress. The traditional water scarcity indices overlook the cumulative impact of climate change, socio-economic patterns, governance, and policies. To bridge this gap, we propose the Integrated Water Strategic Resilience Index (IWSRI), a novel, multidisciplinary index that quantifies water scarcity on the basis of water availability, quality, climate resilience, and socio-political considerations. By integrating hydrological, environmental, and socio-political factors, IWSRI can potentially serve policymakers, researchers, and stakeholders with an interdisciplinary tool for strategic water resource planning. This study outlines the theoretical and mathematical foundations of IWSRI, highlighting its ability to enhance decision-making in transboundary water management, disaster preparedness, and sustainable development. The application of IWSRI is particularly relevant for regions facing severe water stress and political instability, where water availability is both an environmental and security challenge. MENA countries, Israel, Turkey, Qatar, and the UAE possess high water resilience due to solid infrastructure and good governance, while Yemen, Syria, and Libya possess low resilience, driven by conflict and poor management. Egypt, Iran, and Algeria demonstrate moderate resilience due to potential in water management policy. In this respect, while emphasizing its broader applicability as a global tool for assessing water scarcity resilience, this research applies the IWSRI to the MENA region, as its climate, socio-political instability, and regional water stress make it a relevant case study to test its overall efficacy.

## Introduction

Since the late 1980s, when water scarcity became a serious environmental issue involving several countries worldwide, a variety of indicators have been developed in order to facilitate the assessment of the status of water scarcity on a global scale. Consequently, publications on water scarcity assessment have increased in the last two decades, showing a growing interest from a research perspective on the intensification of the problem related to water scarcity. As Liu et al.^1^ have shown, relatively straightforward water scarcity indicators were built between the late 1980s and early 2020s. The indicators have been criticized as focusing solely on water availability and not on the broader socio-economic, environmental, and institutional requirements for a proper assessment of water scarcity. To meet these limitations, there has been a growing shift toward more multidimensional and comprehensive paradigms. Reflecting this trend, a total of 1,682 papers were published from 1970 to 2024 on the topic “water resilience index” based on the Scopus database. There has been a sharp rise in research activity in the last five years: 97 papers in 2020, 142 in 2021, 214 in 2022, 312 in 2023, and 582 in 2024. The rising trend indicates the greater emphasis around the world on water resilience in the context of climate change, sustainability, and resource management.

It is worth considering that climate change is often cited in many studies as one of the leading causes of water stress in various areas of the world^2,3^. Indeed, numerous scientific researches certify that global warming, decreasing precipitation, increasing cases of desertification, and the occurrence of unpredictable weather phenomena—which are among the main effects of climate change—are factors of strong environmental instability that contribute to increasing cases of water scarcity in many regions worldwide^4–8^. Similarly, considerable research efforts have been deployed to show how resource scarcity—especially water—can trigger conflicts in different parts of the world^9,10^,Almer et al., 2017). The number of intra‐state water conflicts keeps increasing globally, especially in developing regions, mostly attributed to physical resources scarcity^11,12^.

At the same time, numerous case studies examined across the years suggest that an evident link exists between the rise of climate-related environmental crises, water scarcity, and sociopolitical factors^2,11,13^. However, establishing a direct causal link between hydrological/climatic data and sociopolitical aspects connected with water management has been rather problematic, as their mutual scientific interaction has often been neglected from a research standpoint^2^. In this respect, “it is needed to consider the rationality of the relationship between socio-economic parameters and the water resource parameters. (…) the level of interaction between these factors should be investigated more”^3^. Consequently, the theoretical assumption of this research is that, despite the centrality of climate and expected climate change impact in generating environmental crises and water scarcity, water management and environmental governance cannot ignore a systemic analysis of other non-hydrological factors. In this regard, as theorized by Nobel Prize Winner Elinor Ostrom, sociopolitical factors, including political stability, industrialization, and community commitment, play a key role in laying the foundations for creating efficient, resilient, and reliable water management systems^14^.

**Fig. 1:**
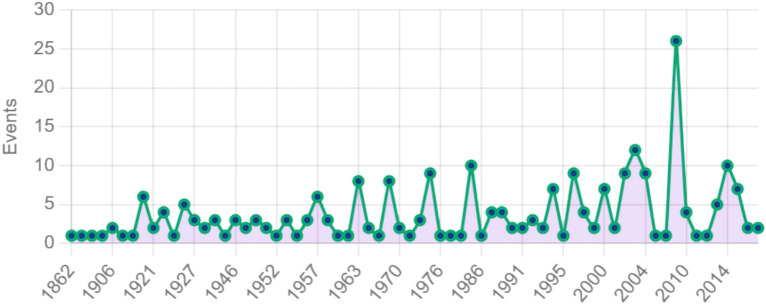
Trends of water peace agreements worldwide.

The relevant lack of a multidisciplinary approach to address water scarcity drives the need to identify new methods of water assessment. These methods involve the adoption of innovative and integrated water indices centred around the analysis of climate change variability and other non-environmentally related factors. The latter are linked to a variety of situations, ranging from human behaviours, water governance, industrialization, infrastructure investments, and transboundary water conflicts. As a matter of fact, water is a much-needed but also highly contested commodity, notably in transboundary river basins where several nations share access to freshwater resources^2,11,13^. In fact, with historic concerns of limited supply and demand by all interested parties, it has often generated tension and conflicts in many cases. In contrast, however, cooperation over shared water resources has far exceeded conflict to develop a category of water peace agreements: treaties and institutional frameworks conceived to drive water management towards sustainability and regional stability. Figure 1 is a representation of the trend in water peace agreements across the world since the 1860s^15^.

In addition, as reported by Cole and Ostrom^14^ and ZamanZad-Ghavidel et al.^3^, sociopolitical factors play a relevant role not only in the management of water as a natural resource but also in the strategic management of water infrastructures for water supply and energy production. In this respect, strategic water infrastructures such as dams, hydropower plants, desalination, and water treatment plants require constant attention from governmental authorities to function properly and efficiently^2,11^. Similarly, the hydro-strategic impact of war must be taken into account, since it typically creates an extremely powerful increase in water stress that, medium and long term, can be the chief cause of alarming cases of water shortage^11,14^.

Some previous research on water scarcity and conflict has drawn on political ecology, often using climate indicators (precipitation decline, temperature rise, evapotranspiration) and environmental stressors (deforestation, vegetation degradation) as predictors^16,17^. However, these studies rarely consider non-environmental factors, and the link between climate-driven sociopolitical crises and water conflict remains insufficiently proven. To address this gap, we developed the Integrated Water Strategic Resilience Index (IWSRI), a composite measure integrating hydrology, climate variability, governance, political stability, infrastructure, and socio-economic adaptability. The MENA region serves as a case study due to its harsh climate, political dynamics, and water stress, though the IWSRI is adaptable for global applications.

While quantitative indices like the Falkenmark Indicator provide measurable benchmarks for hydrological stress, political–ecological perspectives reveal how power dynamics, governance asymmetries, and conflict shape access and equity (e.g.,^18–20^). These approaches are complementary: the former offers empirical rigor for cross-country comparison, while the latter contextualizes outcomes amid socio-political realities. The IWSRI bridges them by operationalizing political–ecological factors—such as governance quality and conflict exposure—into quantifiable proxies, enabling holistic resilience assessment without reducing complexity to numbers alone.

## Methods

Building the Integrated Water Strategic Resilience Index (IWSRI) relies on a quantitative approach that utilizes a mathematical model to assess water scarcity based on environmental, climatic, hydrological, and sociopolitical factors. The process follows a sequential one involving data selection, normalization, weighting, and development of a linear equation for the index determination. To account for the multi-dimensionality of water scarcity, we identified the following essential quantifiable indicators falling under four broad categories:

(i) *Environmental Factors* Groundwater depletion rates, and pollution levels.
(ii) *Climatic Factors* Variability of precipitation, frequency of droughts, and temperature anomalies.
(iii) *Hydrological Factors* Renewable water resources, water demand-to-availability ratio, surface water storage, and transboundary water cooperation.
(iv) *Strategic Factors* Water governance efficiency, infrastructure resilience, political stability, economic adaptability, and desalination capacity.

To validate the robustness of the index, we conducted sensitivity analyses, testing how variations in weights and data sources impact the final outcomes. We also compared the IWSRI outcomes with existing water scarcity indices, such as the Falkenmark Indicator, Water Stress Index, and the Water Poverty Index, to assess consistency and added value.

### Key conceptual definitions

To ensure terminological precision, the following key concepts are defined as the foundation for the IWSRI methodology:

- *Water Scarcity* A multidimensional condition arising from insufficient renewable freshwater relative to demand, exacerbated by climatic variability, population growth, and inefficient management^21^.
- *Water Security* Reliable access to adequate, safe water for human needs, ecosystems, and economic activities, resilient to shocks like drought or conflict^22^.
- *Water Resilience* The capacity of water systems to absorb disturbances (e.g., climate extremes, geopolitical instability), adapt through governance and infrastructure, and maintain core functions^23,24^.

### Key non-environmental indicators—the relevance of sociopolitical factors

Resilience broadly refers to the ability to absorb shocks while maintaining core functions^24^. In water management, it denotes a system’s capacity to anticipate, adapt to, and prevent water stress through strategic planning and long-term measures^23^. Conventional water scarcity assessments often overlook the combined role of climatic and sociopolitical factors. This study integrates governance, political stability, adaptive capacity, and geopolitical vulnerability alongside environmental indicators. These dimensions are operationalized using standardized datasets: the World Bank’s Worldwide Governance Indicators (WGI), the ND-GAIN Country Index, and the Ecological Threat Report’s conflict exposure score. Although simplified proxies, they enable cross-country comparability and quantitative integration within the model.

Countries with strong governance—supported by robust legislation and regulatory frameworks—are best positioned to prevent water overexploitation and ensure equitable allocation^2,25^. Conversely, political instability, corruption, mismanagement, and lack of long-term water planning can lead to inefficient delivery, poor infrastructure maintenance, and conflicts over access^5,25,26^. Transboundary water treaties are also crucial, as shared rivers, lakes, and aquifers require diplomatic cooperation for sustainable water security^19,26^. Governments that adopt strategic measures—such as investing in alternative sources, revising pricing, and incentivizing efficient irrigation—enhance systemic resilience^2^.

It is interesting to note that politically consolidated stability countries usually get foreign investment and are high-income. Therefore, in addition to political stability, one relevant indicator of possible strength of a water system could be associated with the economic ability of a country, i.e., as evidenced by its GDP per capita and nationwide economic development. Interestingly, as highlighted by Gomez et al.^2^ and Farinosi et al.^5^, wealthier nations have more to invest in water infrastructure, sustainable management, and technological innovations, whereas poor nations typically have outdated or inadequate systems.

Infrastructure quality and technology are closely linked to political stability and water management^2,5,25^. Well-maintained networks, modern treatment plants, and diversified infrastructure—such as desalination, rainwater harvesting, wastewater reuse, efficient irrigation, and groundwater recharge—reduce losses, ensure safe water access, and increase resilience to climatic shocks^11^. Conversely, outdated systems, high leakage, and limited treatment capacity can worsen scarcity, even in regions with ample renewable water resources.

The IWSRI, a composite tool for assessing water security, emphasizes both climatological/hydrological variability and sociopolitical factors. Yet, most traditional indices—such as the Green–Blue Water Scarcity Index, QQE Indicator, Water Poverty Index, and Falkenmark Indicator—focus primarily on environmental conditions like rainfall or desertification, often overlooking governance, infrastructure efficiency, and industrial capacity. This highlights the need to integrate sociopolitical indicators alongside environmental metrics for a complete assessment of water security.

The GBWSI, a widely used tool for assessing water availability, focuses on climatic and hydrological factors rather than sociopolitical ones, including geopolitical or conflict-related elements. By distinguishing blue water (surface and groundwater) from green water (soil moisture), it provides a detailed picture of water scarcity and climate change impacts. However, it does not account for sociopolitical factors, limiting its comprehensiveness.

The QQE Indicator, widely used to assess water sustainability, focuses on climatic and environmental factors, measuring water quantity (surface and groundwater), quality (pollution and usability), and environmental flow requirements^27,28^. While effective in capturing physical water stress and human impacts, it omits sociopolitical factors such as governance, political stability, and industrialization, which also shape water availability^12,29–31^. The IWMI similarly emphasizes climate and consumption metrics.

Specifically, it establishes water availability in relation to demand since it considers agricultural utilization, economic capacity, and institutional management as significant parameters to determine if water resources are being effectively utilized or not^31–33^. By chance, IWMI Indicator captures water stress due to environmental factors well but fails to capture policy inefficiencies or destabilizing effects of sociopolitical and geopolitical implications, with a deep consequence on water management in a sustainable manner. Other traditional indices vary in that the IWSRI aims at a multi-disciplinary capturing of water scarcity, combining hydrological data into socio-political and institutional indicators.

This model does not just measure physical water availability but also the strategic capacity of a region to control, respond to, and withstand environmental and geopolitical pressure. With climate change altering precipitation patterns, intensifying droughts, and threatening ecosystems, the index records the broader socio-economic impact of water scarcity on agriculture, industry, and infrastructure. It also evaluates local community and governance resilience in responding to these interruptions, positioning IWSRI as a holistic tool to quantify water resilience in an increasingly unstable world context^4,8^.

### The theoretical use of systemic hydro strategy theory

A new water index covering climate, environmental, and sociopolitical factors is based on multi-scale and systemic examination of the country’s water management policy. For this purpose, our viewpoint is that the optimal theoretical paradigm for designing the IWSRI is the Systemic Hydro Strategy Theory (SHST). The latter, drawing inspiration from a variety of theories, has been built ad hoc to fit theoretical conceptualization of IWSRI and its overall research objective.

At the core of SHST’s theoretical vision is Elinor Ostrom’s theory of polycentric governance, which challenges traditional, centralised, and top-down approaches to water management. Ostrom demonstrated that effective water governance emerges from multi-level institutional arrangements, where local, national, and transboundary actors interact dynamically to shape access and sustainability^14,34^. Ostrom’s approach is essentially based on optimising water governance through agreements at various levels, involving many local, regional, and official actors.

“*When the world we are trying to explain and improve, however, is not well described by a simple model, we must continue to improve our frameworks and theories so as to be able to understand complexity and not simply reject it.*”^34^,665).

Water crises are a product of a complex system of factors involving human and social indicators as well as climatic aspects. Therefore, while recognising the importance of climate and hydrological data, we incorporate Ostrom’s theory as we believe we need a new, more comprehensive framework for assessing the water resilience^14,34^. Consequently, Ostrom’s theory is crucial for the IWSRI, as it underscores the need to evaluate non-environmental indicators such as institutional adaptability, participatory decision-making, and the extent to which water governance systems empower stakeholders. As a matter of fact, a purely hydrological index would overlook the governance asymmetries that lead to inefficient distribution, resource mismanagement, and systemic vulnerabilities in water security.

The hydro-hegemony framework by Zeitoun^20^ and Warner^19^ complement Ostrom’s model by highlighting how power asymmetries shape water governance. Water is viewed not only as a resource but as a strategic asset that can foster cooperation or provoke conflict. In this light, the IWSRI incorporates both physical scarcity and political dimensions, measuring institutional strength, policy effectiveness, and geopolitical dynamics, that influence transboundary water management and national water resilience.

From a systemic perspective, which is central for the definition of an alternative and innovative water index centred around the assessment of resilience, Socio Hydrology, pioneered by^35^, offered critical insights into how human-water interactions evolve over time, creating feedback loops that shape long-term resilience^35,36^. They believe that water management is strongly influenced not only by environmental factors but also by interactive dynamics based on human behaviour. He advocates the creation of alternative methods of hydro-strategic assessment based on the interactions that humans, both as individuals and as communities, have with water systems^35^:

“*Current approaches to studying water sustainability challenges lack explanatory and predictive power because of the inadequate treatment of the two-way dynamic feedbacks between human and water systems. Inadequate explanatory power gives rise to paradoxes, which frustrate efforts to resolve problems in societally relevant ways*.”^35^

Water stress is not a static condition. Rather, in addition to technical indicators related to climatology and hydrology, water stress is dynamically linked to and influenced from other factors such as urbanization, economic shifts, technological innovations, and social adaptation mechanisms^35,36^. Such dynamic scenario underscores the need for an index that captures not just the present state of water resources with a climatic and hydrological focus but, on the other hand, also the adaptive capacity of a system to respond to environmental and socio-political disruptions^11,20^.

### A review of existing indices

Because of the interdependence and complexities of water resources, there is a pressing need for a more holistic framework to assess water resilience. This is especially true under the scenario of climate change, rapid population expansion, and more frequent water-related hazards, where traditional frameworks fail to account for the full range of impacts. To transcend this, the newly proposed Integrated Water Strategic Resilience Index (IWSRI) incorporates several existing indices within a single consistent framework to derive a balanced and resilient index of water resilience.

The IWSRI combines six established indices to provide an aggregated measure of water resilience: the Water Availability Index (per capita resources), Water Quality Index (contamination and treatment), Governance & Policy Index (institutional effectiveness), ND-GAIN Country Index (climate exposure and adaptive capacity), Overall Ecological Score (environmental pressures), and Socioeconomic Resilience Index (economic and social adaptability). Together, they offer a multi-dimensional framework for assessing and enhancing water resilience globally.

Water scarcity measures assess the resilience of a water system—such as a watershed, city, or country—to withstand and recover from stresses including climate change, drought, pollution, and supply disruptions. These indices evaluate the capacity to absorb, adapt to, and recover from pressures affecting water quantity, quality, infrastructure, governance, ecosystems, and disaster risk. Among the most widely used tools is the Falkenmark water stress indicator, which estimates per capita freshwater availability^21^. Similarly, Khoshoei et al.^37^ developed a Multivariate Water Supply Index (MWSI) based on vegetation water demand to assess supply sustainability.

The MWSI is calculated from input parameters such as precipitation, surface water volume, and groundwater volume and can be estimated using Eq. (1):

1 $${MWSI}_{t}={W}_{1}\times {P}_{t}+{W}_{2}\times {SW}_{t}+{W}_{3}\times {GW}_{t}$$

where, ${MWSI}_{t}$ is the multivariate water supply index at time *t*, ${P}_{t}$ is the precipitation at time* t* in mm, ${SW}_{t}$ is the surface water at time *t* in MCM, ${GW}_{t}$ is the ground water at time *t *in MCM, and ${W}_{1}$, ${W}_{2}$, and ${W}_{3}$ are the entropy derived weights for precipitation, surface water, and ground water, respectively.

Sun et al.^38^ formulated a quantitative analysis framework for Urban Water Supply System Resilience (UWSSR) and highlights adaptive management in urban water supply systems to address external changes and highlights the importance of stakeholder involvement in decision-making. Alessa et al.^9^ proposed the Arctic Water Resource Vulnerability Index (AWRVI) as a tool for Arctic communities to assess their vulnerability-resilience in terms of water resource changes and community resilience, i.e., water infrastructure, social capital, and ability to perceive changes in water supply as per Eq. (2):

2 $$AWRVI=\left[{AWRVI}_{physical}+{AWRVI}_{social}\right]/2$$

where AWRVI value ranges between 0 and 1, and the lower value is indicative of highly resilient and the higher value is indicative of more vulnerable. Kuruppuarachchi et al.^39^ proposed a Climate Resilience Index (CRI) for quantifying the resilience of home gardens. The analysis identifies the most important variables influencing the resilience, viz., soil and water conservation practices, presence of woody trees, and species richness, that can be manipulated to enhance the level of resilience. CRI is the weighted average of three components represented by Eq. (3):

3 $${CRI}_{l}=\frac{\sum_{1}^{3}{N}_{k}\times {C}_{k}}{\sum_{1}^{3}N}$$

where: ${N}_{k}$ is the number of home gardens in the sample for the specific component (k), ${C}_{k}$ is the weight, and $N$ is the total number of home gardens.

Liu et al.^1^ introduced a method to evaluate the environmental implications of freshwater use with a focus on the three impact categories: human health, ecosystem quality, and resources. Eqs (4, 5 and 6) show the mathematical formulas to build the Quantity and Quality of Environmental (QQE) water scarcity indicator:

4 $${S}_{qqe}={S}_{quantity}(P)/{S}_{quality}$$

5 $${S}_{quantity}=\frac{BWF}{BWA}=W\times R/(BWR-EFR)$$

6 $${S}_{quantity}=\frac{GWF}{BWR}$$

where *S*_*qqe*_ is the blue water scarcity index, a synoptic indicator for representing water scarcity by considering the quantity of water, ${S}_{quantity}\left(P\right)$ is the water scarcity by quantity, ${S}_{quality}$ is the water scarcity by quality, *BWF* is blue water flow, *BWA* is blue water availability, *W* is water withdrawal, *R* is precipitation in a region, *BWR* is blue water resource, *GWF* is green water flow, and *EFR* is environmental flow requirement.

The Water Availability Index (WAI) is calculated based on per capita annual renewable freshwater resources (billion cubic meters) relative to national population^40^. Water quality is assessed through key physical, chemical, and biological parameters that determine its suitability for human and ecological use. Physical indicators include temperature and turbidity. Chemical parameters comprise pH, dissolved oxygen (DO), biochemical oxygen demand (BOD), chemical oxygen demand (COD), electrical conductivity (EC), total dissolved solids (TDS), and water hardness. The presence of heavy metals—such as lead, mercury, arsenic, and cadmium—as well as salinity, organic compounds (e.g., tannin and lignin), pesticides, and herbicides further defines overall water condition and potential risks to ecosystems and human health (Water Quality by Country, 2025).

Governance refers to the institutions and processes through which power is exercised, including government selection and oversight, policy formulation and implementation, and the relationship between the state and citizens. The Worldwide Governance Indicators (WGI) assess governance performance across more than 200 countries for the period 1996–2023, based on six dimensions: Voice and Accountability, Political Stability and Absence of Violence/Terrorism, Government Effectiveness, Regulatory Quality, Rule of Law, and Control of Corruption^22^ (Table 1).

**Table 1:**
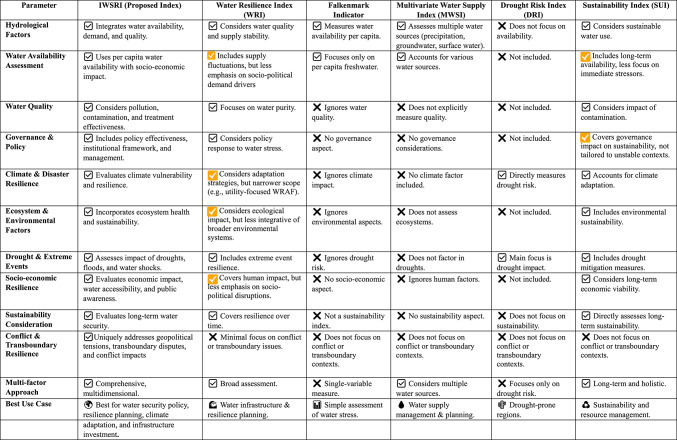
Comparison of the proposed IWSRI index framework with existing indices.

The ND-GAIN Country Index quantifies countries’ vulnerability to climate change as well as their ability to adapt. Where vulnerability is assessed across six sectors of food, water, health, ecosystems, human habitat, and infrastructure in relation to exposure, sensitivity, and adaptive capacity. The index presents a general scenario of risks together with preparedness and assists in identifying where there is most need for adaptation. The equation for calculating ND-GAIN Score^41^ is shown in Eq. (7).7

The Ecological Threat Report (ETR) calculates ecological threats using a collection of environmental, social, and economic indicators that propel the exposure of nations and regions to conflict and instability^42^. The report includes the parameters such as Ecological Degradation, Climate Change, Water Stress, Food Insecurity, Population Growth, Governance and Societal Resilience, and Conflict and Instability.

The Socioeconomic Resilience Index (SERI) measures a community’s capacity to withstand economic, social, and environmental shocks. It integrates four dimensions: economic resilience (diversity, employment, income distribution), social resilience (and education, health, social capital), infrastructure resilience (energy, water, communications systems), environmental resilience (resource management and disaster preparedness)^43^.

The literature review highlights the need for a more comprehensive framework that integrates all critical parameters to accurately assess water resilience. Although this study relies on open-access datasets, data availability and update frequency vary, particularly in conflict-affected countries such as Yemen, Libya, and Syria, where monitoring capacity is limited. To address these gaps, we applied data normalization, cross-source verification, interpolation, and sensitivity analyses, which show that country rankings remain generally stable under different weighting and completeness scenarios. Nonetheless, IWSRI results should be interpreted in context and complemented with locally validated data where possible. The datasets used are reported in Table 2.

**Table 2:** Comparison of various performance indices in the literature for MENA region.

Country	Water Availability Index (2001) (Ref.^40^, and ^45^)	Water Quality Index (2025) (Ref.^46^)	Governance & Policy Index (2023) (Ref. ^22^)	ND-GAIN Country Index (2024) (Ref.^41^)	Overall Ecological Score (2024) (Ref. ^42^)	Socio-economic Resilience Index (2013) (Ref.^43^)
Kuwait	4	73.1	51.42	55.2	2.46	7.5
UAE	18	66.1	95.28	60.3	2.55	7.5
Yemen	98	Not listed	0	36.2	1.67	0
Syria	1799	Not listed	3.3	38.5	1.74	5
Libya	80	Not listed	5.19	43	2.39	7.5
Iraq	2050	57.3	8.49	43.6	1.82	5
Lebanon	821	56.6	6.13	44.7	4.4	5
Egypt	733	54.8	41.98	47.6	2.82	5
Algeria	301	62	27.36	48.7	2.97	7.5
Iran	1488	68.3	14.15	50.6	2.69	5
Jordan	78	63.5	64.62	52.3	2.14	5
Tunisia	332	61.1	39.15	53	3.68	5
Morocco	781	49.2	50	53.4	2.33	7.5
Oman	182	63.5	62.26	53.9	2.67	8
Bahrain	61	64.9	74.53	54.8	2.52	2
Turkey	2289	61.6	41.51	56.5	2.86	7.5
Saudi Arabia	69	60.6	78.77	57.6	2.42	5
Qatar	32	71.5	85.85	59.6	2.72	8
Israel	231	81.7	83.49	62.1	3.34	9

The IWSRI formula is a weighted arithmetic mean of the six factors, and through this approach, each factor contributes to the resulting index. Scaling up the sum by dividing by 6 provides an equilibrated composite score so that the index can be compared. This structure is applied to:

(i) *Capture Multidimensionality* The six parameters cover hydrological, environmental, climatic, and socio-political dimensions, avoiding the restrictive nature of traditional indices (e.g., Falkenmark Indicator, per capita water availability only).
(ii) *Ensure Simplicity and Interpretability* The linear combination is computationally easy and allows policymakers to understand how each parameter contributes to the final score.
(iii) *Allow Flexibility* The weights (*W*_*1*_ to *W*_*6*_) enable tailoring to local or contextual needs, e.g., emphasizing governance in politically sensitive regions or water quality in regions with pollution concerns.

To combine these various indices into a single formula, raw data of each parameter are normalized to the same range (e.g., 0 to 10) in order to compare them. The IWSRI has been conceptualized as a composite index for assessing water resilience by including six key parameters: Water Availability Index (WAI), Water Quality Index (WQI), Governance & Policy Index, ND-GAIN Country Index, Overall Ecological Score, and Socioeconomic Resilience Index (SERI). The expression for the general calculation of IWSRI is presented in Eq. (8):

8 $${\mathrm{IWSRI}} = \left( \begin{gathered} W1 \times {\mathrm{Water}} {\mathrm{Availability}} {\mathrm{Index}} + W2 \times {\text{ Water}} {\mathrm{Quality}} {\text{Index }} \hfill \\ + W3 \times {\mathrm{Governance}} \& {\mathrm{Policy}} {\mathrm{Index}} + W4 \times {\mathrm{NDGAIN}} {\mathrm{Country}} {\mathrm{Index}} \hfill \\ + W5 \times {\mathrm{Overall}} {\mathrm{Ecological}} {\mathrm{Score}} + W6 \times {\mathrm{Socioeconomic}} {\mathrm{Resilience}} {\mathrm{Index}} \hfill \\ \end{gathered} \right)/6){ }$$

where W_1_, W_2_,…,W_6_ are the weights of the parameters.

The parameter-wise average weight for each parameter is calculated and lowest, highest and average weights are considered for the calculation of IWSRI. The generalized formula for calculating average weight is:

9 $${W}_{p} =\frac{\sum_{i=1}^{N}{P}_{i}}{Count of non-NaN values}$$

where *P*_*i*_ is the value of the parameter *P* for the *i*^*th*^ country, *N* is the total number of countries. Count of non-NaN values: The number of valid (non-NaN) entries for the parameter *P*. This may be less than *N* if there are missing values (e.g., NaN).

Though the IWSRI relies on average weights to enable cross-country comparability, the architecture is cognitively constructed to be contextually recalibrated. Where specific dimensions (e.g., governance, climate resilience, or infrastructure) define resilience dynamics in a region, users have the option to employ adapted or regionally specific weighting schemes. Sensitivity analyses (Fig. 8) ensure rankings are relative under different weighting, affirming robustness without compromising methodological flexibility.

Across MENA countries, average weights are: Water Availability (2.62), Water Quality (4.40), Governance & Policy (4.60), ND-GAIN (5.77), Ecological Score (3.56), and Socio-economic Resilience (6.55). The minimum and maximum weights are 2.62 and 6.55, respectively. Applying average, minimum, and maximum weights produces consistent trends in the Integrated Water Strategic Resilience Index (IWSRI) (Fig. 8). Country rankings remain stable across weighting scenarios, confirming the robustness and methodological reliability of the IWSRI.

### Advantages of this method

- Comprehensive and Holistic Approach

Other indicators look at one characteristic of water resilience, at times without regard to the complex set of interactions between water availability, quality, governance, and environmental impacts. The combination of multiple parameters ranging from water availability and quality to governance, environmental risks, and socio-economic resilience provides a more complete picture by the IWSRI.

(b) Climate Change Adaptability and Governance.

The inclusion of the ND-GAIN Country Index and Governance & Policy Index allows the IWSRI to take into account the adaptive capacity and vulnerability of an area, which are most critical in determining long-term water resilience to climate change.

(c) Ecological Threat Consideration

Total Ecological Score incorporates various forms of environmental risks including deforestation, desertification, climate change, and water scarcity. Ecological consideration of this type is needed for understanding general environmental stresses on water resources not necessarily captured by general measures like the Water Availability Index. Regarding indicators in the Ecological Threat Report, the IWSRI also measures likely tensions regarding environmental degradation, food shortages, and water shortages, which are also worthwhile to consider in counting water resilience in vulnerable areas.

(d) Socioeconomic Resilience

With the inclusion of Socioeconomic Resilience Index (SERI), IWSRI can quantify the economic and social capacity of a region to recover and respond to water shocks. Here, socioeconomic resilience is applicable in water-based regions where water is used for agriculture, industry, and domestic uses, and an overlooked dimension in traditional indices. This parameter summarizes the capacity of a community to adapt to water stress and its use of available resources, injecting a measure of resilience that is central to water sustainability.

## Results

**Fig. 2:**
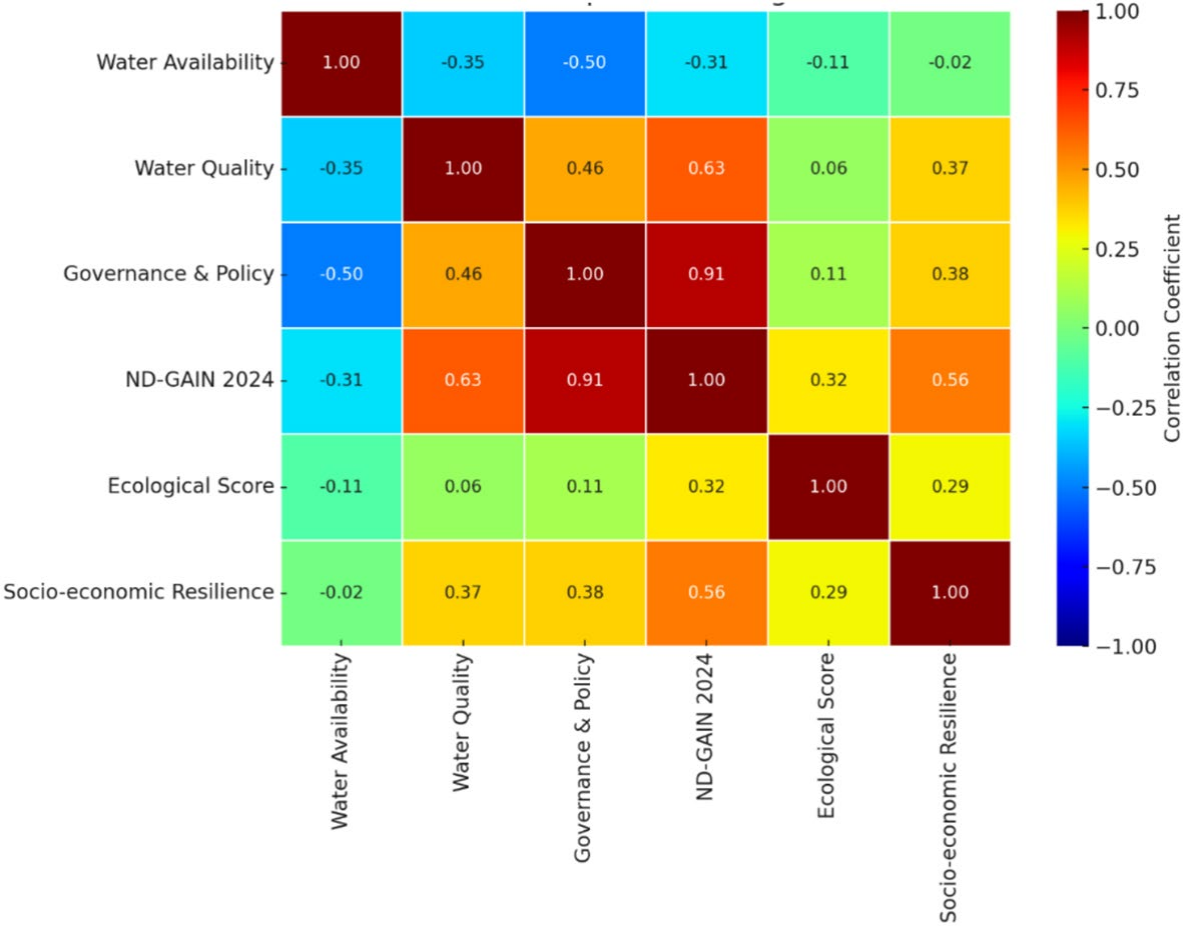
Heatmap of the parameters used for calculating the IWSRI.

A correlation heatmap of the parameters used for calculating the IWSRI, shown in Fig. 2, gives significant correlations between various socio-political and environmental metrics. We found the most significant positive correlations between the ND-GAIN Country Index and the Governance & Policy Index, indicating that better-governed countries possess better climate resilience and adaptation policy-making capabilities irrespective of their physical water endowment. Similarly, Water Quality also shows a high positive correlation with Governance and ND-GAIN Index, implying that better-governed nations have cleaner water and improved climate-related resilience.

Moderate positive correlation exists between Ecological Score and Socio-economic Resilience and between Water Availability and Ecological Score, implying that nations with more sustainable ecosystems are more socially and economically resilient. However, the map also highlights poor or near-zero correlations between Water Availability and measures of governance or resilience, which shows that access to water does not necessarily mean more effective governance or societal resilience. In particular, the countries with scarce renewable water resources, like the UAE and Qatar, are still among the highest socio-economic resilience and governance ranks, indicating the influence of wealth, industrialization, water governance, policy, and innovation in compensating for environmental limitations.

The ISWRI heat map of various nations in the MENA region is compared to check the country-wise water resiliency. The greater the value of IWSRI, the more resilient the country is, i.e., it has applied proper and sufficient policies, infrastructure, and resource usage to combat water problems. On the other hand, a low IWSRI value shows low resilience, and it refers to countries that are water-stressed, mismanaged, or have stressors from outside affecting their water availability. The map illustrates the differences in water resilience using a red-to-green color scheme so that dark green (index value 10) represents the most resilient country.

**Fig. 3:**
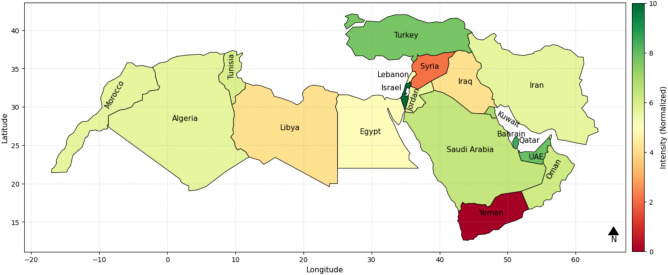
IWSRI index heat map with all six considered parameters.

Based on the calculated IWSRI Index, Fig. 3 shows that countries like Turkey, Israel, Qatar, UAE are represented in dark green, showing high water resilience with good infrastructure and long-term strategic vision. On the other hand, nations like Yemen and Syria are represented in red and orange, which shows low resilience in the form of ongoing conflict, lack of water, and inefficient management. Nations such as Egypt, Iran, Jordon, and are represented in the middle group, showing moderate resilience with areas for improvement.

**Fig. 4:**
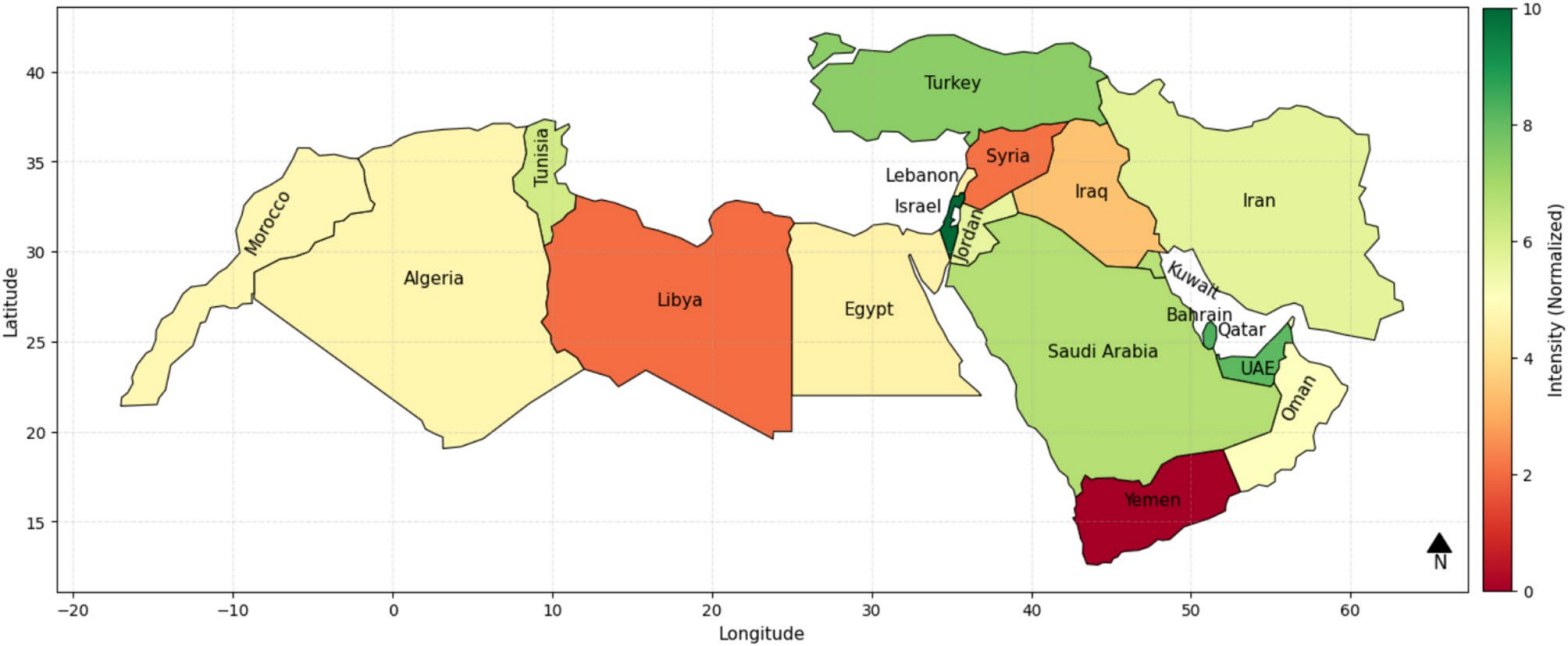
IWSRI index heat map without considering the Socio-economic Resilience Index parameter.

Figure 4 presents an IWSRI heatmap of the MENA region, indicating the water resilience of various countries based on hydrological, governance, and environmental parameters. The Socio-economic Resilience Index parameter was excluded in order to examine the effect of these other parameters separately from socio-economic status. It shows Israel, Turkey, Qatar, and UAE as the strongest, in dark green, reflecting highly developed water management, even when socio-economic power is not considered. Libya, Syria, and Yemen, in red and dark orange, present some of the lowest IWSRI values, reflecting enormous water-related issues, exacerbated by political instability and war in some cases. Algeria, Iran, Iraq, Egypt, and Saudi Arabia are shaded in yellow and light green, indicating a moderate resilience with varying water infrastructure capacity and policy performance.

**Fig. 5:**
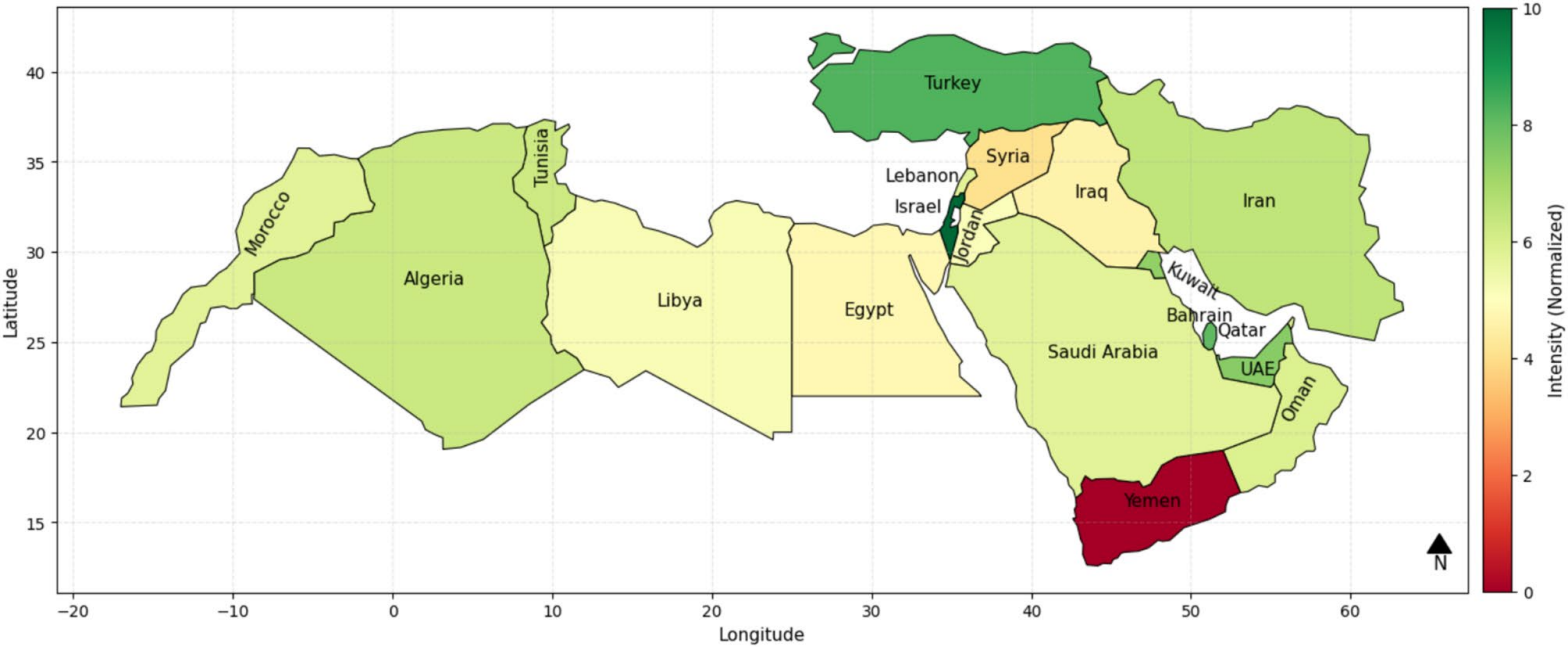
IWSRI index heat map without considering the Governance & Policy Index parameter.

Figure 5 illustrates an IWSRI heatmap of the MENA region, where water resilience of nations is depicted across hydrological, environmental, and socio-economic indicators. Governance & Policy Index has been excluded to emphasize physical and social resilience factor contributions separated from governance. It shows that Israel, Turkey, Qatar, Kuwait, and UAE remain the best-performing nations, highlighted in dark green indicating their high infrastructural and natural resilience. At the other end, Yemen stays dark red, indicating bare exposure in water systems regardless of governance. Tunisia, Algeria, Iran, and Morocco are in different shades of green, denoting strong physical and socio-economic resilience. Libya, Egypt, and Iraq are in light yellow and orange colors, denoting moderate resilience, possibly lacking infrastructural development or environmental development despite any governance assistance.

**Fig. 6:**
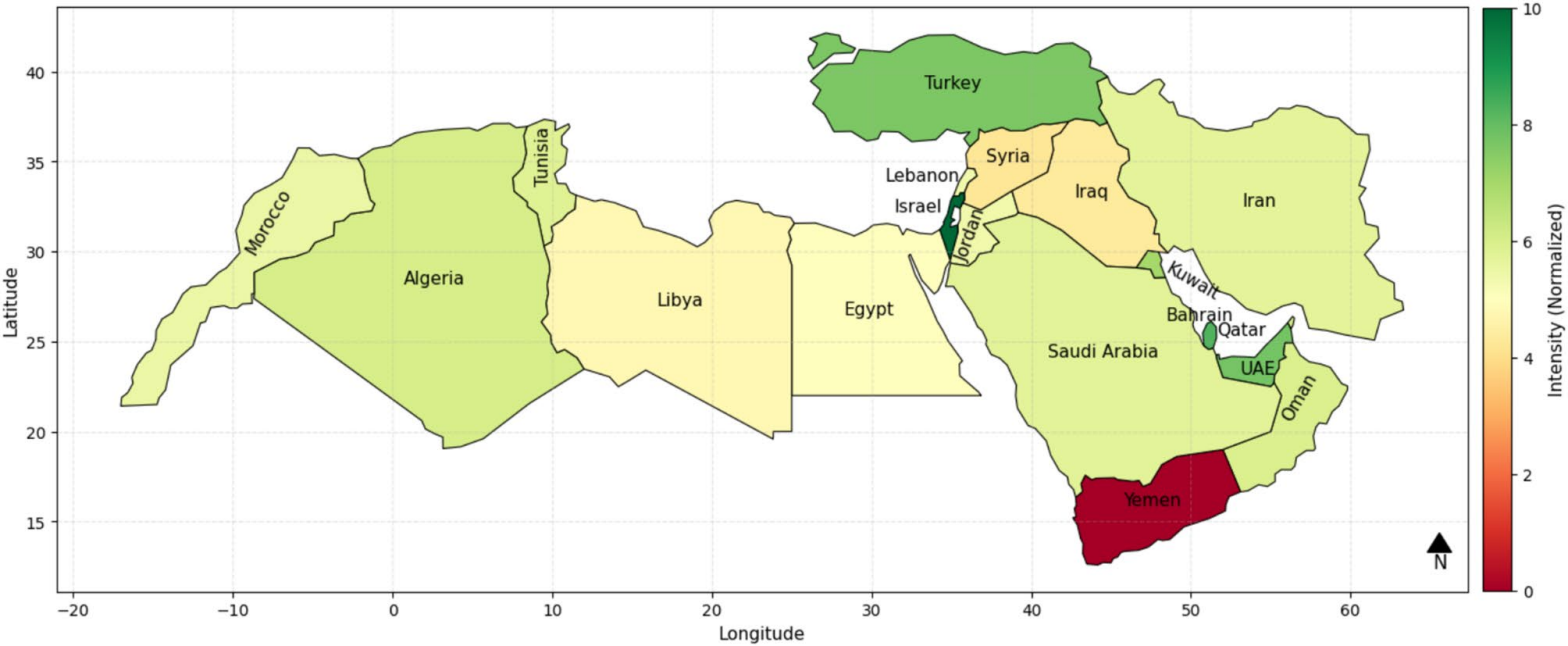
IWSRI index heat map without considering the ND-GAIN Country Index parameter.

Figure 6 is an IWSRI heatmap of the MENA region illustrating water resilience in countries by governance, socio-economic, and ecological indicators. The ND-GAIN Country Index was excluded to identify the impact of factors independently of climate vulnerability and adaptive capacity. It shows Turkey, Israel, Qatar, and the UAE in dark green color, representing good water management policy, infrastructure levels, and governance. Conversely, Yemen is dark red, and highly elevated water insecurity, and conflict, poor mismanagement, and low levels of infrastructure. Iran, Algeria, Morocco, and Saudi Arabia belong to the category of medium resilience, which translates to yellow to pale green meaning restricted success at preventing water issues but where room for improvement exists.

**Fig. 7:**
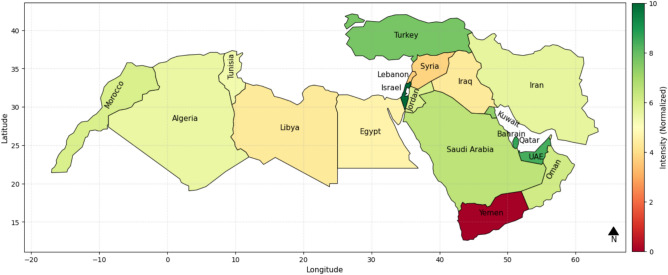
IWSRI index heat map without considering the Overall Ecological Score parameter.

**Fig. 8:**
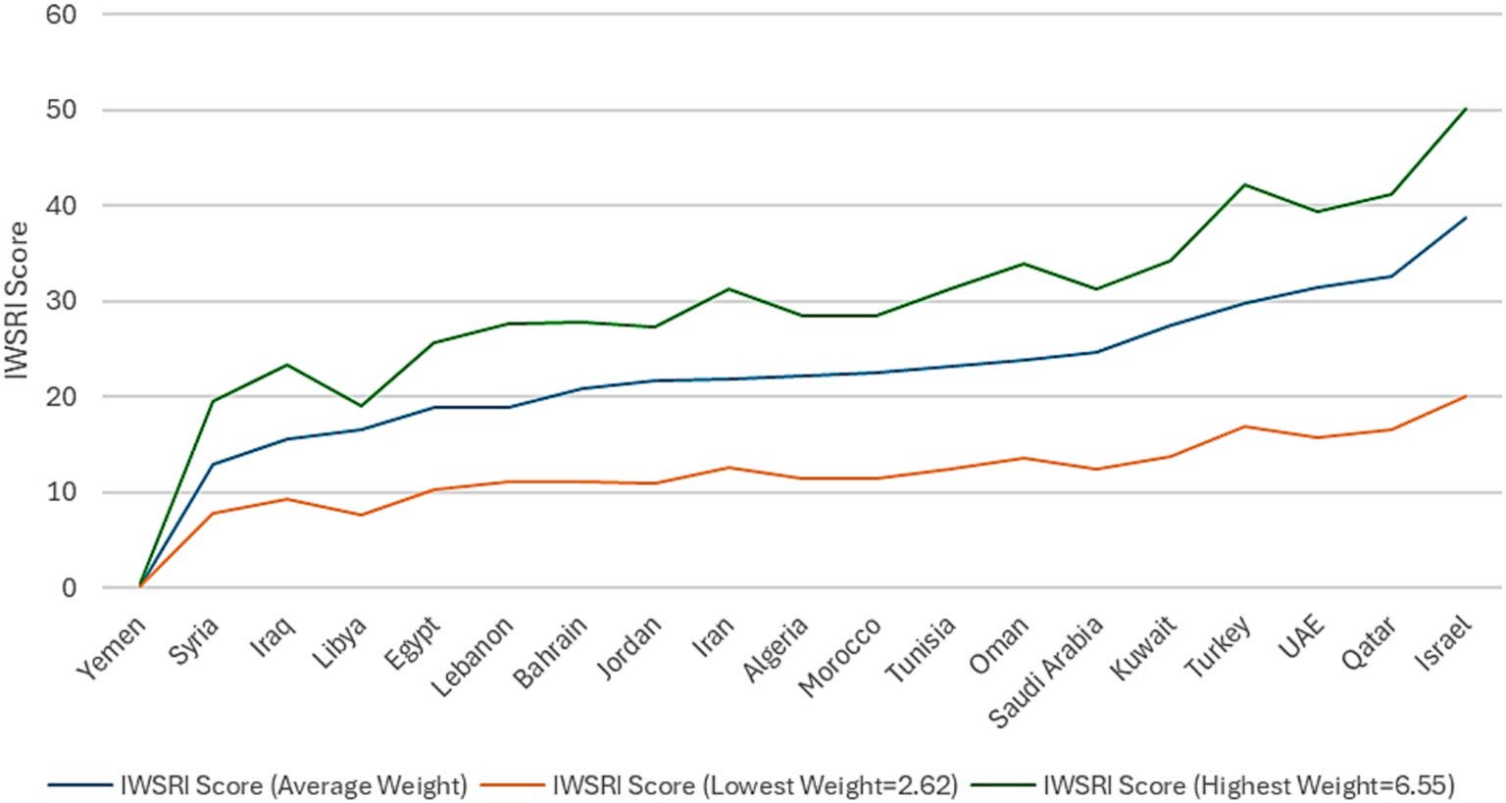
IWSRI comparison with different weights.

Figure 7 illustrates an IWSRI heatmap of the MENA region, and countries are presented based on water resilience based on hydrological, governance, and socio-economic drivers. The Overall Ecological Score was eliminated so that the relative contribution of the factors could be examined independently from ecological conditions. It shows the countries such as Turkey, Israel, Qatar, and UAE as dark green, where high water resilience is achieved by means of farsighted policy, technology expenditure (e.g., desalination, reuse), and institutional arrangements. Yemen is dark red, suggesting an extreme water insecurity most likely to result from conflict, bad government, and the absence of proper infrastructure. Libya, Iraq, and Syria fall within the lower-to-moderate resilience grouping (orange to yellow) by proxies of political instability or as water infrastructure-based vulnerability. All the other countries, including Morocco, Iran, Saudi Arabia, and Egypt, light green to yellow hues, showing moderate resilience with scope for strategic improvement (Fig. 8).

The IWSRI heat maps highlight significant disparities in water resilience across MENA. Israel, Turkey, Qatar, and the UAE rank high (dark green) due to strong governance, advanced infrastructure, and effective water management, despite severe physical scarcity in some cases. In contrast, Yemen, Syria, and Libya rank very low (red/dark orange), reflecting scarcity, conflict, mismanagement, and weak infrastructure. Countries such as Egypt, Iran, Algeria, Iraq, Morocco, Tunisia, and Saudi Arabia show medium resilience (yellow/light green), indicating partial progress but persistent governance, infrastructure, or environmental gaps. Sensitivity analysis shows that removing key parameters like ND-GAIN or ecological scores does not significantly alter rankings, suggesting that resilience is primarily driven by infrastructure quality, policy effectiveness, and technological investment^44^.

Table 1 compares the proposed IWSRI with five existing frameworks: the Water Resilience Index (WRI), Falkenmark Indicator, Multivariate Water Supply Index (MWSI), Drought Risk Index (DRI), and Sustainability Index (SUI). The comparison highlights IWSRI’s broader scope, particularly in integrating policy and governance, institutional capacity, and water management—dimensions largely absent in Falkenmark, MWSI, and DRI. IWSRI also provides a comprehensive assessment of climate vulnerability and resilience, whereas DRI focuses mainly on drought risk and others omit climate factors. Environmental and ecosystem components are fully embedded in IWSRI, only partially addressed in WRI and SUI, and largely absent in the remaining indices.

One of the striking features of IWSRI is its assessment of drought and unusual events such as floods and water shocks, a feature where some indices such as DRI and WRI exhibit strength but not some such as Falkenmark and MWSI. Further, socio-economic resilience involving water supply, economic impact, and societal consciousness is well covered in IWSRI, WRI, and SUI but neglected by Falkenmark, MWSI, and DRI. On the sustainability dimension, IWSRI and SUI are specifically designed to measure long-term water security and economic sustainability as opposed to other indices that are either oblivious of sustainability or approach it lightly.

The IWSRI assessment highlights the close link between climate stress, conflict, and weak water governance in the region. Yemen, Libya, and Syria rank lowest, as severe climate pressures and prolonged conflict have devastated infrastructure and service delivery. Iraq and Algeria perform slightly better, though both face drought and fragile governance; Iraq benefits from the Tigris and Euphrates rivers, while Algeria’s relative stability supports resilience^1,12^. Lebanon ranks mid-range: despite high rainfall and historically strong management, political and economic crises have weakened its system. Overall, the contrast between resilient and fragile states underscores the decisive role of political stability and institutional strength in shaping water security under climatic stress^7,37^.

Iran, Egypt, Jordan, Tunisia, and Morocco fall under the moderate resilience grouping. Despite being faced with significant climate stresses, these states have promoted tremendous developments in water policy management. Moreover, the fact that these countries are not necessarily engaged in active conflict and are mostly situated in the arid regions shields key water infrastructure from attack or destruction by hostile forces, thereby providing them with an enhancement of their resilience. However, issues such as population growth, transboundary water tensions, and resource overuse still pose risks to long-term stability. Very relevant countries from a demographic standpoint, such as Egypt and Iran, experience a sharp water demand. While Iran has access to multiple sources of surface water bodies (Karoon and Zayandeh-Roud Rivers) and underground reservoirs, Egypt is undoubtedly more at risk as it can only count on a single abundant source of water supply: the Nile River^38,47–50^ .

In contrast, the Persian Gulf monarchies, Turkey, and Israel rank high in the IWSRI due to technological advancement, financial capacity, and stable governance. Despite extreme aridity (except Turkey), they offset natural scarcity through strategic investment in desalination and strong institutional capacity—an advantage clearly captured by the IWSRI^51,52^. Saudi Arabia, the UAE, Qatar, Oman, and Kuwait achieve high resilience through large-scale desalination, ensuring reliable supply even during drought. Israel further strengthens resilience with world-leading wastewater recycling. By reducing dependence on limited freshwater resources, and in cases like Qatar and Kuwait relying almost entirely on desalination, these states demonstrate how long-term planning and technology can secure water resilience in hyper-arid, geopolitically complex regions^35,53^.

As shown in the IWSRI assessment (Fig. 3), Israel, Qatar, the UAE, and Turkey display high water resilience despite aridity and regional instability. Israel stands out: its resilience is driven not by favorable climate conditions but by advanced water technologies and strategic governance. Large-scale desalination supplies up to 80% of its drinking water, while over 85% of wastewater is recycled for agricultural and industrial use. Decentralized and well-protected infrastructure further ensures continuity during conflict. Israel thus illustrates how technological investment and institutional capacity can offset structural vulnerabilities and elevate resilience within the IWSRI framework^48,54^.

Turkey is one of the "water resilience powers" of the IWSRI due to the fact that it is politically stable, well-governed, and endowed with a vast amount of water resources. Unlike most MENA conflict-affected nations, Turkey possesses secure institutions and long-term water planning^15,55^. With the hosting of the headwaters of rivers like the Euphrates and Tigris, it has invested heavily in infrastructure, especially the Southeastern Anatolia Project (GAP), in favor of agriculture and energy. Its robust hydropower sector generates clean energy while being sustainable. This combination of natural endowment and strategic management makes Turkey water secure even amidst regional unrest and increasing climate variability^19^.

## Discussion

Unlike widely used models such as the Falkenmark Water Stress Indicator, Water Security Index (WSI), and Water Poverty Index (WPI)—which focus mainly on water availability, access, and economic conditions—the IWSRI integrates socio-political dynamics and conflict as core determinants of water resilience. While translating political and institutional processes into quantitative proxies may risk oversimplification, the IWSRI is intended as a diagnostic tool that identifies systemic resilience patterns rather than a definitive measure of governance performance. By incorporating mismanagement, instability, war, and transboundary tensions, it addresses a critical gap in water resilience research. Traditional indicators, particularly Falkenmark and WSI, remain centered on physical availability and service access, overlooking governance failures and hydro-political conflict.

Although not included in Table 1 due to its broad and general nature, the Water Security Index (WSI) remains a useful indicator of long-term water availability, governance, and vulnerability. Similarly, while the Water Poverty Index (WPI) incorporates socio-economic factors, it largely overlooks the security dimension, particularly how military conflict intensifies water stress by damaging infrastructure, displacing populations, and militarizing resources. With conflicts increasingly affecting regions such as MENA, there is a clear need for updated indices that integrate socio-economic and political fragility. Countries like Yemen, Syria, and Libya—where war, climate stress, and weak governance intersect—offer critical cases to evaluate the effectiveness of the IWSRI framework^3,53^.

The Green–Blue Water Scarcity Index (GBWSI) is a widely recognized tool for measuring water availability, particularly in relation to climate variability. However, it excludes socio-political variables, limiting its applicability in conflict-affected or politically unstable contexts such as Lebanon, Syria, Libya, or Yemen^12,37^. The index does not account for infrastructure destruction, governance breakdown, forced displacement, or the militarization of water resources—factors that critically shape water access in war-torn regions. In areas like the Middle East and parts of Africa, scarcity stems not only from climatic stress but also from geopolitical rivalries and conflict. While GBWSI effectively captures hydrological pressures, it overlooks the water-conflict-security nexus that the IWSRI framework seeks to address^23,27,28,38^.

The Quantity-Quality-Environmental Flow Requirement (QQE) Indicator, while widely used, focuses mainly on climatic and environmental factors and ignores socio-political dynamics. In contrast, the IWSRI incorporates geopolitical instability, transboundary conflicts, governance, and infrastructure impacts on water access. As shown in Fig. 3, water scarcity in much of MENA results not only from climate variability but also from mismanagement, conflict, and resource control. While QQE is useful for environmental sustainability, IWSRI, as shown in this research, provides an integrated assessment of water resilience that addresses both climate and socio-political risks, making it better suited for high-risk regions^3,53^.

The IWMI Indicator, while widely applied, focuses on hydrological and economic factors but overlooks the effects of conflict and war on water security^28,38^. By contrast, the IWSRI integrates climatic and socio-political variables, including infrastructure destruction, displacement, and transboundary conflicts. In politically unstable regions, water access depends not only on climate and economy but also on governance breakdown and military control. IWSRI thus provides a more strategic framework for assessing water resilience where conflict and climate intersect.

### Water scarcity as a social construct

Water scarcity, assessed here through economic, political, and environmental lenses, also has profound social dimensions. Leyla Mehta’s work on India highlights that scarcity is not merely “too little rain” but is shaped by politics, institutions, and social practices^56^. Governments, experts, and NGOs often “naturalize” scarcity, privileging urban and industrial users while marginalizing the poor. Social hierarchies—caste, class, and gender—determine who bears the burden, with women and lower-caste communities disproportionately affected. Technologies and markets, such as deep tubewells and tanker services, can exacerbate inequalities under the guise of efficiency. Mehta reframes scarcity as a political and social condition, produced as much by policy and infrastructure choices as by climate, highlighting persistent issues of power, entitlement, and justice^18,57^.

In the Middle East, Schuetze Hussein et al.^58^ show that in Jordan and across MENA, “scarcity” is politically constructed. State and donor narratives frame shortages as natural, masking allocation choices and power dynamics. Elites use scarcity discourse to secure aid, justify mega-projects, and maintain patronage, while refugees are often blamed to deflect attention from over-extraction and inequitable distribution. Similarly, Allouche^59^ highlights North Africa’s “hydraulic mission,” where centralized, technocratic interventions—dams, transfers, and desalination—consolidate authority and direct investment, often sidelining equity and local rights. Scarcity narratives thus transform complex socio-ecological realities into apolitical management issues.

The IWSRI has been specifically applied to the MENA region due to its desert-like nature, transboundary water dependence, and vulnerability to both climate change and geopolitical tensions. MENA provides a case of significant water resilience challenges, such as desertification, aquifer depletion, dust storms, and land degradation. However, the IWSRI is designed for global application. Its methodology enables adaptation across diverse hydrological and political settings, from South Asia to Latin America, making it suitable for conflict zones, regions with agricultural stress, or countries advancing hydro-diplomacy. It offers a comprehensive tool to assess how effectively nations manage water under environmental and strategic pressures.

## Conclusion

Water insecurity is often seen as a climatic or hydrological issue, but global crises show that socio-political and economic factors are equally critical. Climate variability and droughts are compounded by weak governance, institutional fragmentation, and conflict. Addressing water challenges requires more than hydrological solutions. The Integrated Water Strategic Resilience Index (IWSRI) offers a multi-disciplinary approach, combining hydrological, environmental, economic, and socio-political indicators to assess water resilience by linking physical stressors to governance, institutional stability, conflict intensity, and adaptive capacity.

Unlike other classical water scarcity metrics, which to a great extent depend on physical water availability, the IWSRI acknowledges that water security has close interdependencies with climatic resilience, political stability, social equity, economic resilience, and resource management. The index, by incorporating these elements, aims to offer a more accurate and actionable framework to stakeholders. Without an integrated approach, it is likely that water scarcity will continue to fuel instability, exacerbating conflict cycles. Strengthening hydro-political governance, investing in adaptive infrastructure, and fostering inclusive water-sharing agreements are essential for ensuring long-term water resilience and regional stability.

We believe that water indices modeled on the IWSRI play a critical role in establishing standardized, yet adaptable, tools for assessing the resilience of water systems on a global scale. In this paper, we have used the MENA region as a case study to demonstrate the relevance of a multidimensional water index that takes into account qualitative and quantitative aspects. However, we believe that the application of the IWSRI is global in scope, as the assessment of the resilience of a water system in light of environmental and climatic phenomena and socio-economic indicators has implications for all countries of the world, with different outcomes depending on the water policies adopted by local authorities.

### Limitations

One of IWSR’s key challenges lies in the variability and inconsistency of data availability across different regions, particularly in fragile or conflict-affected areas such as Yemen, Libya, or Syria, where institutional capacity and monitoring infrastructure are often lacking due to warfare and political instability. This makes rigorous assessments and cross-country comparisons challenging to standardize. Additionally, the IWSRI might face temporal limitations due to the inconsistent availability and update frequency of its component indices. Many relevant datasets, especially socio-political or ecological indicators, are released at irregular intervals or lag behind current conditions. This temporal mismatch can hinder real-time assessments, reduce responsiveness to emerging crises, and limit the accuracy of forecasting water system resilience over time. As a result, while the IWSRI remains a robust diagnostic tool, we acknowledge that its application must be critically interpreted in light of local socio-political realities and data limitations.

In addition, the interpretation of socio-political factors within the IWSRI framework presents an added layer of complexity. To be specific, governance mechanisms, power differentials, institutional trust, and local conflict patterns are qualitative and context-specific in nature, so it is difficult to measure them in a constant and standardized index such as the IWSRI. Such a scenario may reflect ambiguity in how such factors are weighted or read. This ambiguity, if not adequately addressed, could lead to oversimplifications that may not reflect the complex political economy of water access or water governance. For instance, in regions like the MENA, where governance is often contested and fragmented, these interpretive challenges become particularly pronounced, limiting the accuracy and policy relevance of resilience assessments.

While we propose to adopt the IWSRI with a global approach, we also recognize that another challenge arises when attempting to interpret IWSRI data in relation to global scenarios, such as climate change projections, international trade dynamics, or shifting geopolitical alliances. These macro-level drivers regularly impinge on local water systems in unforeseen manners. This heavily impacts resource availability, infrastructure investment, and transboundary cooperation dynamics that are important parts of the IWSRI. As such, the static or localized moments captured by the IWSRI may fail to capture the cascading impact of global disruptions, including supply chain crises, regional conflict, or international policy changes, that can quickly shift a nation’s water resilience.

This article develops and tests the IWSRI in the MENA region, leveraging rich empirical data and observable hydro-political dynamics to evaluate the index’s coherence. While designed for global application, the IWSRI requires context-specific adaptation: each basin or country needs tailored variables and recalibrated weights reflecting local governance, infrastructure, climate, and socio-economic conditions. Its modular framework, with transparent normalization and sensitivity testing, ensures interpretability across contexts. MENA serves as a proof-of-concept, with validity elsewhere achieved through careful recalibration and documentation.

## Data Availability

The datasets used and/or analysed during the current study are publicly available from the following sources: - Total Population by Country (2025): World Population Review database, available at [https://worldpopulationreview.com/](https:/worldpopulationreview.com) - Water Quality by Country (2025): World Population Review’s water quality rankings, available at [https://worldpopulationreview.com/country-rankings/water-quality-by-country](https:/www.worldpopulationreview.com/country-rankings/water-quality-by-country) - Governance & Policy Index (2023): World Bank, available at [https://www.worldbank.org/en/publication/worldwide-governance-indicators/interactive-data-access](https:/www.worldbank.org/en/publication/worldwide-governance-indicators/interactive-data-access) - Socio-economic Resilience Index (2013): PreventionWeb, available at [https://www.preventionweb.net/files/31553\_socioeconomicresillianceindex2013ma.pdf](https:/www.preventionweb.net/files/31553_socioeconomicresillianceindex2013ma.pdf) All data were accessed between February and March 2025.
